# Cholinergic dysfunction in occupational manganese exposure

**DOI:** 10.1016/j.neuro.2025.103313

**Published:** 2025-09-03

**Authors:** T. Noah Hutson, Susan Searles Nielsen, Natalie Senini, John O’Donnell, Hubert P. Flores, Tamara Hershey, Joel S. Perlmutter, Anil Kumar Soda, Stephen M. Moerlein, Zhude Tu, Michael Kasper, Lianne Sheppard, Brad A. Racette, Susan R. Criswell

**Affiliations:** a Department of Neurology, Barrow Neurological Institute, 240 W Thomas Rd, Phoenix, AZ 85013, USA; b Department of Neurology, Washington University School of Medicine, 660 S. Euclid Ave, St. Louis, MO 63110, USA; c Department of Radiology, Washington University School of Medicine, 660 S. Euclid Ave, St. Louis, MO 63110, USA; d Department of Psychiatry, Washington University School of Medicine, 660 S. Euclid Ave, St. Louis, MO 63110, USA; e Program in Physical Therapy, Washington University in St. Louis, St. Louis, MO, USA; f Program in Occupational Therapy, Washington University School of Medicine, 660 S. Euclid Ave, St. Louis, MO 63110, USA; g Department of Neuroscience, Washington University School of Medicine, 660 S. Euclid Ave, St. Louis, MO 63110, USA; h Program in Physical Therapy, Washington University School of Medicine, 660 S. Euclid Ave, St. Louis, MO 63110, USA; i Department of Environmental and Occupational Health Sciences, University of Washington, School of Public Health, 3980 15th Ave NE, Seattle, WA 98195, USA; j Department of Biostatistics, School of Public Health, University of Washington, 3980 15th Ave NE, Seattle, WA 98195, USA; k School of Public Health, Faculty of Health Sciences, University of the Witwatersrand 27 St. Andrews Rd, Parktown 2193, South Africa

**Keywords:** Manganese, PET, Cholinergic, Biomarkers, Neurotoxicology

## Abstract

**Background and objective:**

Excessive exposure to manganese (Mn) produces a clinical syndrome of parkinsonism and cognitive impairment. However, our understanding of the mechanisms of Mn neurotoxicity remains limited. This study aimed to evaluate the relationships between Mn exposure, cholinergic function, and cognitive impairment in exposed workers.

**Methods:**

We assessed brain cholinergic function using vesicular acetylcholine transporter (VAChT) radiotracer (-)-(1-(8-(2-[(18)F]fluoroethoxy)-3-hydroxy-1,2,3,4-tetrahydronaphthalen-2-yl)-piperidin-4-yl)(4-fluorophenyl) methanone (VAT) with positron emission tomography (PET) in 21 Mn-exposed workers. We estimated occupational Mn exposure from work histories and the MRI pallidal index. A cognitive control battery consisting of the Verbal Fluency (VF), Letter Number Sequencing (LNS), Two-Back Letter Task (2B), Go-No-Go (GnG), and Simon Task assessed cognitive function. We applied generalized linear models to Mn exposure, voxel-based cholinergic PET, and cognitive control measures, estimating coefficients for cholinergic-mediated associations between Mn and cognitive function. We utilized bootstrapping techniques to validate the mediation coefficients.

**Results:**

Both Mn exposure metrics were associated with low cholinergic VAT binding in the caudate and cortical regions including the precuneus, pars triangularis, pars opercularis, middle temporal lobe, and entorhinal cortex. Regional cholinergic function mediated the relationship between Mn exposure and both the composite cognitive control score (mean of the 5 cognitive tests) [β = −0.661, 90 % confidence interval (CI) −2.130, −0.032] and the individual VF assessment (β = −0.944, 90 % CI −2.157, −0.065).

**Discussion:**

Higher Mn exposure is associated with lower cholinergic activity in multiple brain regions. Cholinergic function also mediates a portion of the relationship between Mn exposure and cognitive control performance. Caudate and cortical cholinergic activity may be a biomarker of early Mn neurotoxicity and represent an important mechanism of cognitive dysfunction in parkinsonian syndromes.

## 1. Introduction

Manganese (Mn) exposure impacts millions of people worldwide from occupational and environmental exposures secondary to industrial emissions and other sources of fossil fuel combustion (Neal and Guilarte, 2013). Mn is also a critical element in electronic vehicle batteries and the resulting massive quantities of battery waste are creating new sources of human exposure. Mn is a neurotoxicant that, in excess, produces a clinical syndrome of parkinsonism and cognitive impairment (Rodier, 1955). Cognitive impairment in Mn neurotoxicity increases in a dose-dependent manner with cumulative exposure and affects performance on tasks that require working memory, attention, concentration, cognitive flexibility, and visual spatial skills (Bowler et al., 2006; Bowler et al., 2015). While these associations are well described in Mn neurotoxicity, the underlying mechanisms are largely unknown.

The cholinergic system is physiologically essential in aspects of locomotion and cognition (Finkelstein et al., 2007). Current models of cognitive decline in idiopathic Parkinson disease (PD) and Parkinson disease dementia (PDD) propose a “dual-syndrome” or “two-hit” effect in which early dopaminergic deficits and later cholinergic deficits create the conditions for the development of cognitive impairment and dementia (Kehagia et al., 2013 Bohnen et al., 2015). As with PD and PDD, previous research in Mn neurotoxicity has focused on the involvement of dopaminergic systems despite evidence that Mn has demonstrable effects on the neuronal cholinergic synapse including choline uptake, choline acetyltransferase (ChAT) activity, acetylcholine release, and the regulation of acetylcholinesterase (AChE) (Finkelstein et al., 2007).

Early in-vivo molecular imaging studies of cholinergic systems in patients with PD and dementia were limited to PET tracers of AChE activity, which provided relatively poor regional specificity. PET imaging of vesicular acetylcholine transporter (VAChT) represents a major advance in imaging of the cholinergic system (Petrou et al., 2014). VAChT is only present in cholinergic cells and is expressed on presynaptic cholinergic terminals, where it appears to be tightly regulated with ChAT, as they share the same gene locus (Berrard et al., 1995). Pre-clinical and initial human studies using PET imaging VAChT radiotracers including (-)-(1-(8-(2-[(18)F]fluoroethoxy)-3-hydroxy-1,2,3, 4-tetrahydronaphthalen-2-yl)-piperidin-4-yl)(4-fluorophenyl)methanone (VAT) demonstrate high brain uptake and excellent selectivity and specificity to human cholinergic nerve terminals (Petrou et al., 2014; O’donnell et al., 2024). This new generation of cholinergic tracers allows for improved assessment of cholinergic dysfunction, as indicated by lower VAT binding, in neurodegenerative conditions including Alzheimer disease (Aghourian et al., 2017) and PD (Slingerland et al., 2024).

The goal of this study is to use [(18)F]VAT PET to measure in-vivo cholinergic function in Mn-exposed workers and examine its association with both Mn exposure and cognitive control performance. We previously observed poorer cognitive control performance in relation to higher cumulative occupational Mn exposure (Al-Lozi et al., 2017). In the present work, we hypothesize higher Mn exposure will be associated with lower regional cholinergic [(18)F]VAT non-displaceable binding potentials (BP_ND_) and subsequent poorer cognitive control performance in Mn-exposed workers. Given the similarities in clinical phenotype between Mn neurotoxicity, PD, and PDD we further hypothesize that underlying cholinergic dysfunction, as measured by VAT BP_ND_, represents an important mechanism in cognitive control dysfunction associated with Mn exposure, i.e., that cholinergic dysfunction mediates at least part of the association between Mn exposure and cognitive control, and thus may provide critical insight into the common pathophysiology of these overlapping conditions.

## 2. Methods

### 2.1. Protocol approvals and participant consents

This study was approved by the Washington University Human Research Protection Office and conducted in accordance with the principles expressed in the Declaration of Helsinki. All participants gave written, informed consent prior to participation.

### 2.2. Participants

All participants (N = 21, two repeat studies for a total of 23 records) were from the United States (U.S.) Midwest and underwent [(18) F]VAT PET between 1/9/2017 and 3/20/2023. We recruited participants from Mn-exposed workers who participated in our previous cohort study (Racette et al., 2017) from three Midwestern shipyards. The 21 Mn-exposed workers included 13 welders and eight workers from the same sites who worked around welding fume (Table 1). Possible worker occupations included or were a combination of the following: electrician, fabricator, fitter, inspector, machinist, millwright, painter/-sandblaster, structural iron/steel worker, and technician. All reachable participants from the original union-based cohort (Racette et al., 2017) were offered participation in this study. Exclusion criteria were history of liver disease, stroke, brain tumor, or other condition that could compromise the neurologic examination; use of anti-parkinsonian medications, neuroleptics, or amphetamines that could affect VAT binding; and age < 18 years, all of which were uncommon.

### 2.3. Clinical assessments

Trained coordinators administered five tasks that assess cognitive control in one or more of its subdomains (e.g., response inhibition, working memory, fluency). These five tasks, described below, are validated measures that are frequently used in the cognitive neuroscience literature and previously used to assess cognitive control dysfunction in this cohort (Al-Lozi et al., 2017).

1. Verbal Fluency (VF): VF assesses response set shifting and fluency by having the participant list as many words as possible that fit a criterion in one minute. We used an age-adjusted total scaled score for this analysis (Mcdowd et al., 2011).
2. Letter Number Sequencing (LNS): LNS assesses verbal working memory span. The participant must use working memory to repeat and rearrange randomly assorted letters and numbers into numerical and alphabetical order. We used the age-adjusted total scaled score as the primary outcome for the LNS (Gabrieli et al., 1996).
3. Two-Back Letter Task (2B): 2B assesses working memory for verbal information. The participant decides as quickly as possible if a stimulus matches the one that appeared two trials earlier, testing both working memory and response inhibition. We used discriminability (mean accuracy rate minus the false positive response rate) as the primary outcome of the 2B test (Braver et al., 2001b).
4. Go-No-Go (GnG): The GnG task assesses the ability to inhibit a prepotent response using verbal stimuli and requires active cognitive control processes such as conflict monitoring. The participant must exercise response inhibition to one of four stimuli as each stimulus appears in random order at fast intervals. We used discriminability as the primary GnG outcome (Braver et al., 2001a).
5. Simon Task (Simon): This task is based on the Simon Effect in which an impertinent spatial stimulus will slow down reaction time to a target stimulus when the correct response button is spatially discordant with the impertinent, distracting stimulus (Wylie et al., 2010). The difference between the reaction time for congruent and incongruent trials was used in our analysis. Difference values were calculated so that lower values were associated with lower performance, thus the direction of scores corresponded to the other four tests.

To create a composite cognitive control summary score, we first calculated z-scores for each prespecified outcome variable for each task above. We then averaged these five z-scores (Al-Lozi et al., 2017). All scores (except the 2B test in which one participant failed to complete) were available for 18 of the 23 records. The remaining 5 participants did not undergo cognitive testing, as the protocol did not initially include the cognitive batteries.

### 2.4. Exposure assessment

All participants completed or updated a validated, structured questionnaire in person at the time of imaging, which included a detailed complete work history (Hobson et al., 2009). Using this information, we calculated cumulative lifetime occupational Mn exposure in mg Mn/m^3^-years, which takes into account both duration (years) and intensity (mg Mn/m^3^) of exposure (Racette et al., 2017). As a secondary measure of exposure we also obtained the MRI T1-weighted pallidal index (PI). Respirable Mn from occupational exposure deposits in the brain and can be detected on T1-weighted MRI, most prominently in the pallidum (Nelson et al., 1993). The T1 MRI PI is a well-established in-vivo marker of Mn exposure where a higher PI value is associated with greater welding exposure(Majewski et al., 2024; Criswell et al., 2012). Both the mg Mn/m^3^-years metric and PI were available for all participants (records), and corresponded with the date of the respective PET scan.

### 2.5. MRI acquisitions

All magnetic resonance imaging scanning was performed on a Siemens Magnetom Prisma 3T scanner (Erlangen, Germany) with a 12-channel head coil. We acquired high resolution three-dimensional (3-D) magnetization-prepared rapid gradient echo (MPRAGE) images on each participant (repetition time [TR]=2400 ms, inversion time [TI]=1000 ms, echo time [TE] = 3.14 ms, flip angle = 8°, 0.9 ×0.9 ×0.9 mm voxels). We used FreeSurfer^™^ (v5.3, Laboratory for Computational Neuroimaging, Charlestown MA) automated atlas-registration and volume segmentation from MPRAGE data to obtain the pallidal region and the previously described white matter reference regions to calculate the MRI PI (Criswell et al., 2019) and [(18)F]VAT binding potential respectively (O’donnell et al., 2024). Volume and segmentation data were reviewed by a single investigator blinded to work history and clinical findings.

### 2.6. PET acquisition

PET was collected with a single Siemens ECAT EXACT HR+ scanner. Three retractable germanium 68 (68Ge) rod sources were used for transmission scans to measure individual attenuation factors. Transaxial and axial reconstructed spatial resolution at slice center were 4.3 mm and 4.1 mm full width half maximum (FWHM) in 3D mode (Brix et al., 1997). [(18) F]VAT was synthesized as previous described (Tu et al., 2015) and between 11.31 mCi - 14.82 mCi was intravenously injected into an arm vein over 30 s. We obtained 3-dimensional (3-D) dynamic PET images for 120 min beginning with radioligand injection, with eight 30 s frames, four 60 s frames, six 120 s frames, and twenty 300 s frames. PET scans were reconstructed with filtered back projection with ramp filter cut-off at the Nyquist frequency and included attenuation, scatter, deadtime, and randoms corrections as described previously (O’donnell et al., 2024).

### 2.7. PET image processing and analysis

#### 2.7.1. Generation of voxel-wise parametric binding potential (BP) images

We segmented each participant’s MR image into volumes of interest (VOIs) using Freesurfer^™^, as described above. The MR images were then co-registered to each participant’s PET images to permit extraction of tissue activity curves (TACs) for each VOI from the set of dynamic PET images. Next, we utilized the white matter reference region to calculate voxel-wise non-displaceable binding potentials (BP_ND_) using Logan analysis applied to TACs from 30 to 120 min post-injection (Logan, 2000). All steps were performed in native PET reconstruction space.

Following estimation of BP_ND_ per voxel, we co-registered parametric PET images to MRI space via a 6-parameter vector gradient linear transformation. The MR co-registered parametric images were then transformed into 711–2B atlas space (anisotropic voxel size 1.2 × 1.5 × 1.5 mm) by a second 6-parameter linear transform to bring all images into comparable space. We selected the 711–2B atlas based on the age distribution of the participants.

#### 2.7.2. Voxel-defined spatial associations between exposure and VAT BP_ND_

A voxel-based analysis was used to identify a map of voxels with robust associations between cumulative Mn exposure and VAChT binding. This newly defined VOI was used to more robustly examine associations between Mn exposure and cholinergic function from a data-driven, whole brain approach. To define this VOI, we first estimated voxel-wise generalized linear models (GLM)s between mg-Mn/m^3^-years and VAT BP_ND_, adjusting for age as a linear term. A mask of brain volume specific to the present 23 PET studies was applied to the parametric GLM maps. We identified individual voxels exhibiting high absolute values of marked regression coefficients ($\hat{\beta }$) and R^2^ values, and low (significant) p-values (Mikhno et al., 2008; Akamatsu et al., 2019). The first threshold, for the regression coefficient, was defined by generating normal distribution parameters, $\hat{\mu }$ (estimated as the mean of $\hat{\beta }$ and $\hat{\sigma }$($\hat{\beta }$ standard deviation), from all voxel GLM values in the masked volume. Voxels with coefficients with an absolute value greater than $\hat{\mu }$ by at least $0.5\hat{\sigma }$ passed to the next threshold. The second threshold, for R^2^ values, was set to 0.5 (voxels with R^2^ > 0.5 passed) and the third threshold, for p-values, was set to 0.05 (voxels with p < 0.05 passed through). This generated a pair of masks for voxels with significant 1) positive or 2) negative coefficients estimated from each GLM. We aggregated VAT BP_ND_ values of the newly defined VOIs (positive and negative β masks) by participant and a final GLM was applied to examine the relationship between cumulative Mn exposure and this aggregated VAT BP_ND_ adjusted for age. As an alternative to our primary exposure variable, we conducted a secondary voxel-wise age-adjusted GLM analysis with PI and aggregated VAT BP_ND_. We utilized these respective masks to ensure a relation between the respective Mn exposure metric and VAT BP_ND_, as this is required for mediation to occur as hypothesized. An association between VAT BP_ND_ and cognitive control performance is also a requirement. However, we did not apply this latter requirement when defining our mask because we had several cognitive control performance variables and these data were available for fewer people.

#### 2.7.3. Cholinergic VAT BP_ND_ as a potential mediator of the association between Mn exposure and cognitive control performance

**Fig. 1.**
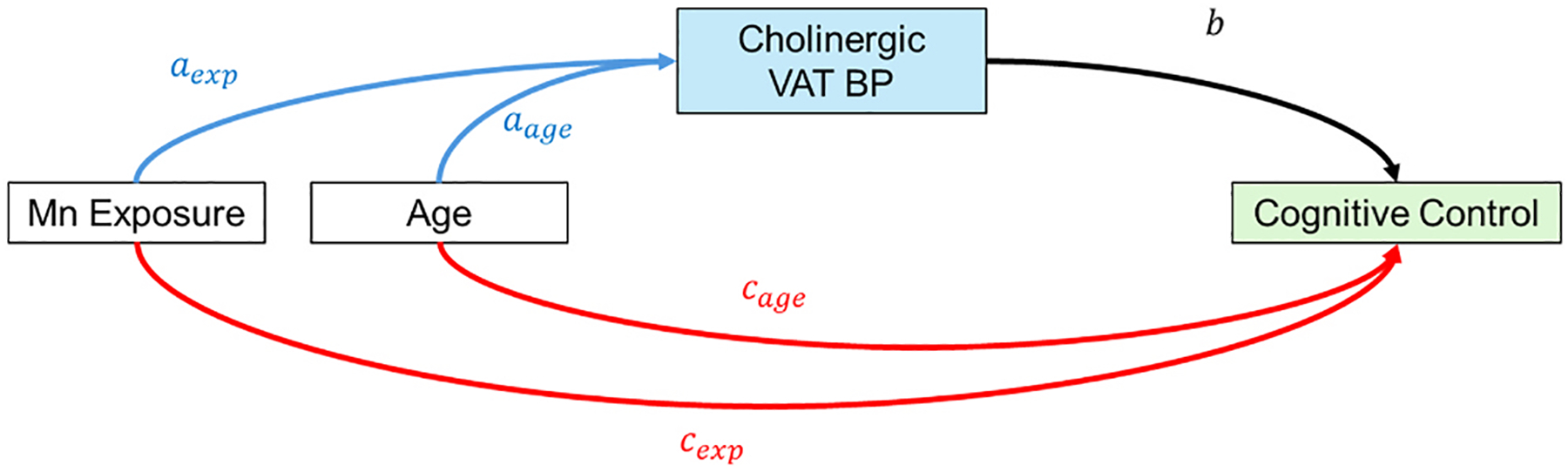
Mediation model from Mn exposure to cognitive control performance through cholinergic VAT BP_ND_ while adjusting for age. Direct pathways of influence from Mn exposure and age on cognitive control function are shown in red (see Eq. (2)), while indirect (VAT BP_ND_ mediated) pathways are represented by the products of the blue and black arrows (also see Eq. (1)). Mn = manganese; VAT = (-)-(1-(8-(2-[(18)F]fluoroethoxy)-3-hydroxy-1,2,3,4-tetrahydronaphthalen-2-yl)-piperidin-4-yl)(4-fluorophenyl)methanone; BP_ND_ = non-displaceable binding potentials.

We modeled the association between the respective Mn exposure metrics and cognitive control performance mediated through cholinergic function (VAT BP_ND_). Using VAT BP_ND_ in the newly defined VOI, we estimated path-specific and total indirect effects of Mn exposure on cognitive control performance through cholinergic VAT BP_ND_ (Fig. 1). We similarly included age in each model due to its strong association with cumulative Mn exposure, VAT BP_ND_, and cognitive control. Specifically, we leveraged the Preacher-Hayes bootstrapping method, generating 5000 bootstrap samples with replacement to determine if VAT BP_ND_ was a path-specific mediator and the combination of VAT BP_ND_ and age (age through VAT BP_ND_) was a total mediator between Mn exposure and the cognitive control summary score (Hayes and Preacher, 2014). This non-parametric approach is optimized for smaller sample sizes, and leverages bootstrapping to define a percentile-based confidence interval of mediation coefficients, allowing for higher probability of statistically robust findings. We then repeated this analysis for each individual cognitive score.

For each bootstrap sample and cognitive control performance, we first estimated the model coefficients of exposure and age in relation to VAT BP_ND_, respectively *a*_exp_ and *a*_*age*_ in the model below:

(1) $${\text{VAT\_BP}}_{\text{NDi}}=\beta_{1}+a_{\text{exp}}•{\text{Exposure}}_{i}+a_{\text{age}}•{\text{Age}}_{i}+\epsilon_{1}$$

Where *VAT*_*BP*_*NDi*_ represents the aggregated binding potential in the newly defined VOI per participant, *β*_1_ represents the intercept term, *Exposure*_*i*_ and *Age*_*i*_ represent the Mn exposure metric and age (both as continuous variables) of each participant, and *ϵ*_1_ represents the error term.

Next, we extracted the coefficient *b* from the following model:

(2) $${\text{Cog}}_{i}=\beta_{2}+c_{\text{exp}}•{\text{Exposure}}_{i}+c_{age}•Age_{i}+b•{\text{VAT\_BP}}_{\text{NDi}}+\epsilon_{2}$$

Where *Cog*_*i*_ is the value of the cognitive control summary score (or individual cognitive test) per participant, *b* represents the connection from cholinergic VAT BP_ND_ to cognitive control performance, and *c*_exp_ and *c*_*age*_, respectively, represent direct effects (those not mediated through VAT BP_ND_) of age and Mn exposure on cognitive control performance.

While some cognitive tests incorporated age into the scaled score, not all tests did. Thus, for our mediation analysis, we included age as a covariate to rule out its influence on VAT mediated relationship between Mn exposure and cognitive control.

We then took the products *a*_exp_ • *b* and *a*_*age*_ • *b* to determine the value of the indirect pathways respectively from Mn exposure and age to cognitive control performance through VAT BP_ND_. We computed these coefficients and their products across the 5000 bootstrapped samples to estimate 90 % confidence intervals (CI) for indirect pathways (Hayes and Preacher, 2014). We used 90 % CIs (one-sided tests) based on the directional hypothesis that cholinergic neurons would be selectively impacted by Mn exposure leading to worse cognitive control performance (negative mediation coefficient). Given our specific directional hypothesis (that the mediation coefficient would be lower than 0), we used a 90 % CI as the probability of type 1 error for the specific single-tailed test for our alternative hypothesis is 95 %. Within the model that we developed, path-specific mediation refers to mediation of the Mn exposure-cognitive control by VAT BP_ND_, while total mediation refers to the sum of the above in parallel with age (mediation by VAT BP_ND_ and age).

MATLAB 2024b was used for voxel-wise and cluster-based statistical analysis of the exposure data, images, and cognitive outcomes.

### 2.8. Data availability

Data from research participants in this study, who authorized sharing of their research data, will be made available to investigators with appropriate expertise and research support after publication of the primary aims of this study. All shared data will be deidentified and will be released in accordance with US regulations.

## 3. Results

### 3.1. Characteristics of participants

Nineteen workers were non-Hispanic White men and two were non-Hispanic White women (Table 1). All had similar education levels, either high school graduate/GED or two years of secondary education. Workers had a relatively wide range of duration of Mn exposure (1.25–41.09 years), but the majority had < 5 years of exposure. Workers had a similar age and sex distribution as compared to the larger cohort (Racette et al., 2017) from which they were recruited (data not shown). Participant cognitive control scores exhibited a wide range of scores, especially for the Simon test (Table 2).

### 3.2. Cumulative Mn exposure and cholinergic binding

**Fig. 2.**
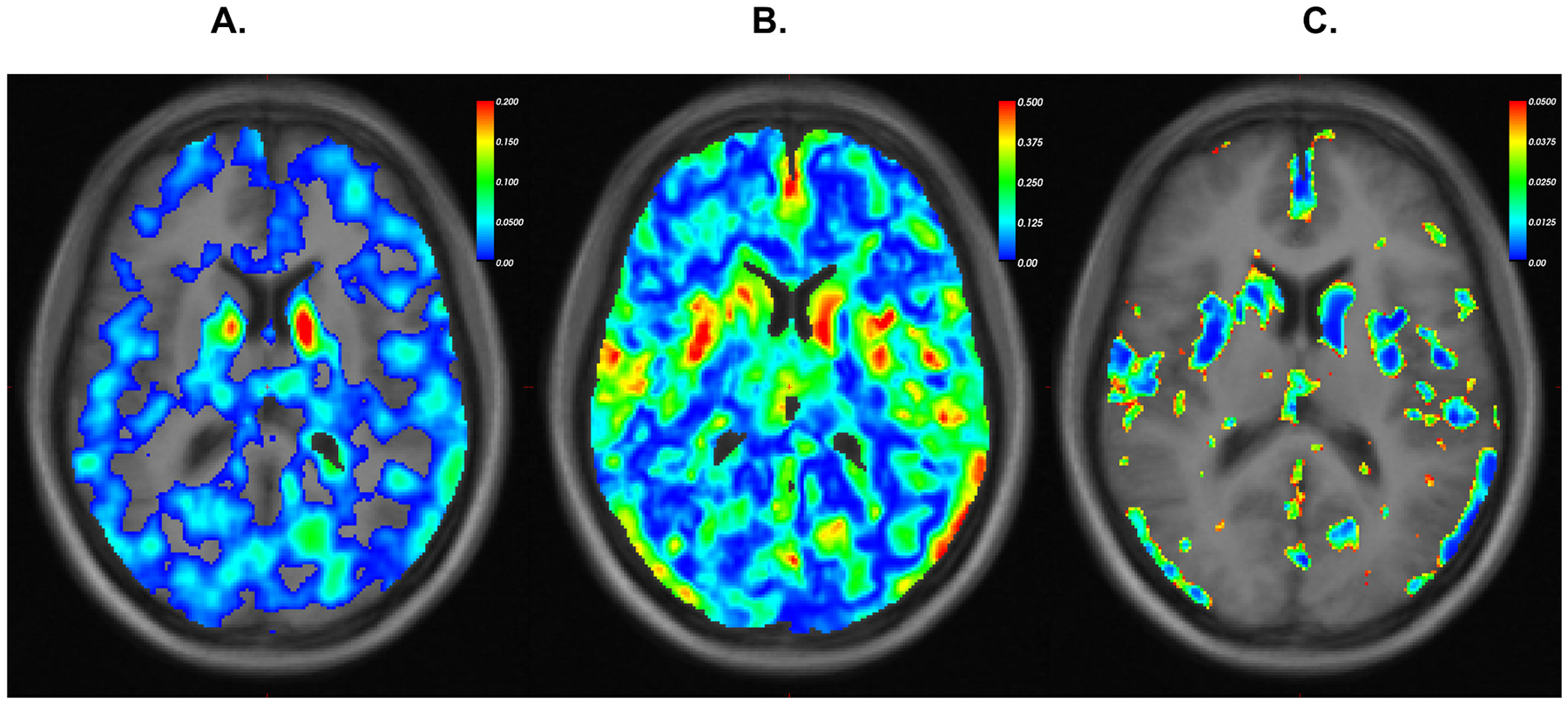
GLM parameter maps (estimated between mg-Mn/m^3^-year and cholinergic VAT BP_ND_ with age adjustment) per voxel on a selected slice depicting values for the three criteria for voxel selection: A. magnitude of the negative beta coefficients; B. r^2^ values; and C. p-values. Voxels selected for final cluster would exhibit high (red or yellow) values for panels A and B while exhibiting low (green or blue) p-values for panel C. GLM = generalized linear models; Mn = manganese; VAT = (-)-(1-(8-(2-[(18)F]fluoroethoxy)-3-hydroxy-1,2,3,4-tetrahydronaphthalen-2-yl)-piperidin-4-yl)(4-fluorophenyl)methanone; BP_ND_ = non-displaceable binding potentials.

**Fig. 3.**
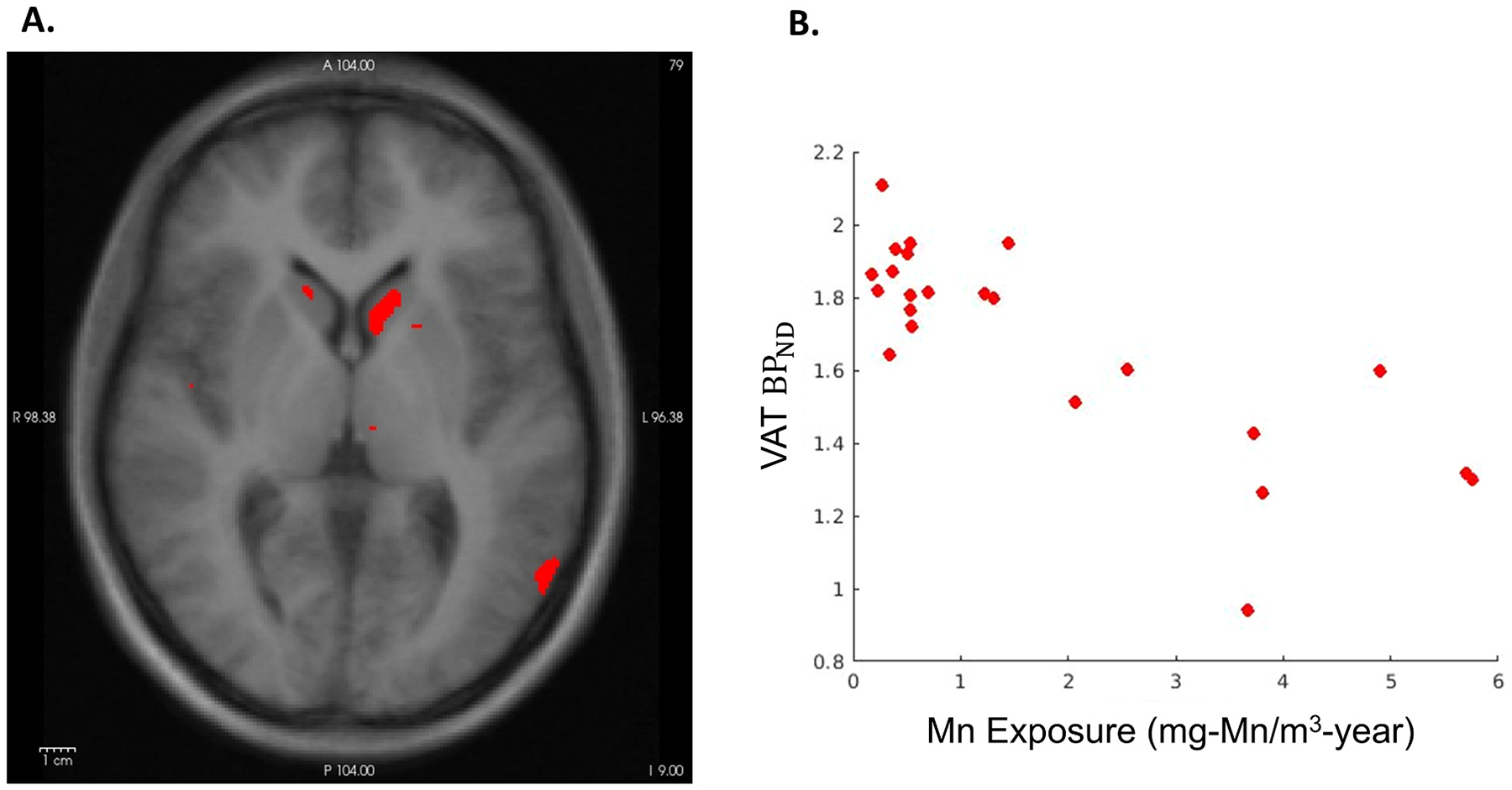
A. Representative slice of a VOI cluster (in red) superimposed on the T1 MPRAGE MR atlas, and B. The association between cumulative Mn exposure (mg-Mn/m^3^-year) and cholinergic VAT BP_ND_ for the full VOI represented in A in red. VOI = volume of interest; MPRAGE = magnetization-prepared rapid gradient echo; Mn = manganese; VAT = (-)-(1-(8-(2-[(18)F]fluoroethoxy)-3-hydroxy-1,2,3,4-tetrahydronaphthalen-2-yl)-piperidin-4-yl)(4-fluorophenyl)methanone; BP_ND_ = non-displaceable binding potentials.

Higher cumulative Mn exposure was associated (β: −0.074, p-value: <<0.001) with lower cholinergic VAT BP_ND_ within the data driven VOI including regions with both high magnitude of β-coefficient/R^2^ and low p-values (Fig. 2). Based on 3171 voxels that met these criteria, the largest concentrations of voxels within the negative β coefficient mask were in the left caudate, with representation in the right caudate, and cortical regions including the precuneus, pars triangularis, pars opercularis, middle temporal lobe, and entorhinal cortex (Fig. 3). The approximated Cohen’s f^2^ effect size of our covariates in the GLM based on the null and model deviances was 3.24, representing a significant explanatory value of cumulative Mn exposure and age on VAT BP_ND_. Regional associations in space and direction of association were largely overlapping when we used PI rather than our cumulative Mn metric. We found no significant direct (positive) voxel-averaged associations between Mn exposure and cholinergic VAT BP_ND._

### 3.3. Mn-exposure, cholinergic binding, and cognitive control – results of the mediation analysis

High cumulative Mn exposures (mg-Mn/m^3^-years) mediated by VAT BP_ND_ were associated in specific ([VAT BP_ND_ & mg Mn/m^3^-years]-β coefficient −0.661, 90 % CI −2.130, −0.032) and total ([VAT BP_ND_ & mg Mn/m^3^-years/age]-β coefficient −0.671, 90 % CI −2.113, −0.032) paths with low cognitive control summary scores (Table 3). We additionally observed VAT-mediated age-adjusted associations in the same direction between cumulative Mn exposure and individual cognitive tests, however, this was only significant for VF ([VAT BP_ND_ & mg Mn/m^3^-years]-β coefficient −0.9443, 90 % CI −2.1574, 0.0651). Point estimates for the direct associations between cumulative Mn exposure and cognitive control summary and individual test scores were also negative but their 90 % CIs included zero.

**Fig. 4.**
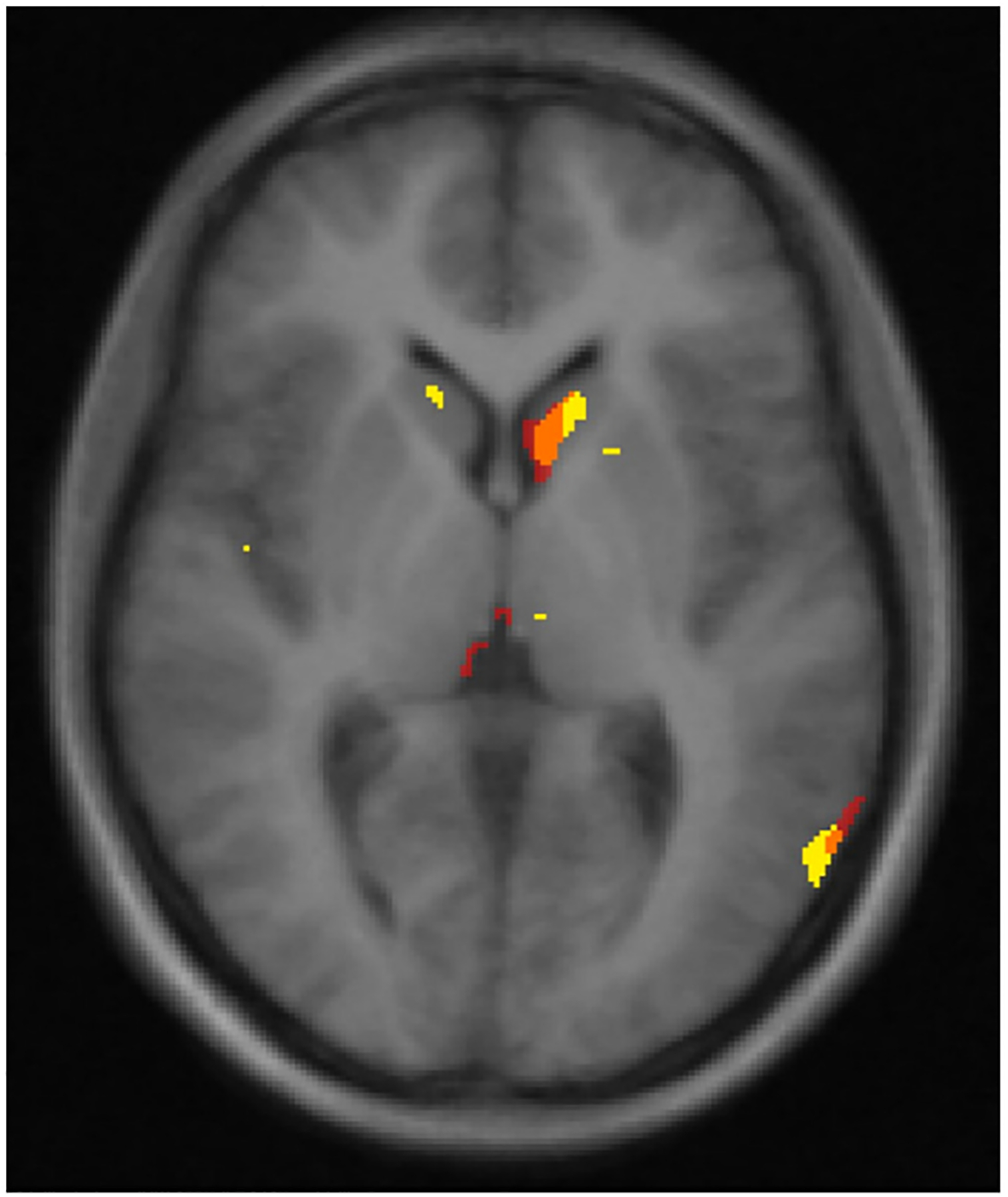
Representative slice of VOI clusters for mg-Mn/m^3^-year/cholinergic VAT BP_ND_ (in red) and PI/VAT BP_ND_ (in yellow) associations superimposed on 711–2B atlas. Areas of overlap are represented in orange. VOI = volume of interest; Mn = manganese; VAT = (-)-(1-(8-(2-[(18)F]fluoroethoxy)-3-hydroxy-1,2,3,4-tetrahydronaphthalen-2-yl)-piperidin-4-yl)(4-fluorophenyl)methanone; BP_ND_ = non-displaceable binding potentials; PI = pallidal index.

In our secondary analysis that used the PI as the measure of Mn exposure, regions exhibiting associations between PI and cholinergic [(18)F]VAT BP_ND_ largely overlapped with those obtained from the cumulative Mn (mg Mn/m^3^-year) analysis (Fig. 4), in addition to small clusters in the insular cortex and posterior isthmus cingulate cortex. Also consistent with the work history-based metric, we found indirect associations in the VAT-mediated age-adjusted relationship between PI and cognitive control performance for specific ([VAT BP_ND_ & PI]-β coefficient −0.288, 90 % CI −0.537, −0.067) and total ([VAT BP_ND_ and PI/age]-β coefficient −0.491, 90 % CI −0.820, −0.142) mediation.

## 4. Discussion

This cross-sectional study investigates cholinergic function and cognitive control performance in relation to occupational Mn exposure and identifies multiple findings illustrating the importance of the cholinergic system in the pathophysiology of Mn neurotoxicity. First, using cholinergic [(18)F]VAT PET, we demonstrate clear associations between Mn exposure and cholinergic activity within the brain. Specifically, higher levels of Mn exposure related to lower cholinergic VAT BP_ND_ in hundreds of voxels throughout the brain, but these associations were most evident in the caudate and cortical regions including the precuneus, pars triangularis, pars opercularis, middle temporal lobe, and entorhinal cortex. Second, leveraging bootstrapping techniques for the small sample size, we detected mediation effects of regional cholinergic function as measured by VAT BP_ND_ in the relationship between Mn exposure and cognitive control performance. The nature of this indirect effect suggests that the relationship between cholinergic function and cognitive control performance may be affected by a region-specific sensitivity to Mn neurotoxicity. These associations were supported using the T1-weighted PI as an alternative measure of Mn exposure with strikingly similar regional results.

Further, we identified these changes in in-vivo cholinergic activity and cognitive performance in a cohort with an estimated mean time-weighted Mn exposure of 0.14 mg Mn/m^3^. This estimated exposure is substantially lower than the US Occupational Safety and Health Administration (OSHA) Permissible Exposure Limit for Mn of 5 mg Mn/m^3^ (Occupational Safety And Heath Administration Osha, 2020) and more consistent with the American Conference of Governmental Industrial Hygienists (ACGIH) time-weighted average threshold limit value for inspirable Mn of 0.1 mg Mn/m^3^ (American Conference of Governmental Industrial, 2013). Given that we observe in-vivo changes in cholinergic function and lower cognitive performance scores in subjectively normal workers at levels slightly above the ACGIH standard, our study provides additional support for reducing the OSHA Permissible Exposure Limit to the ACHIG threshold limit value. Cholinergic [(18)F]VAT uptake may represent an in-vivo biomarker of early cognitive or even subclinical Mn neurotoxicity, although the latter would require further study.

Occupational Mn exposure results in Mn deposits throughout the brain with some of the highest concentrations in the basal ganglia, frontal cortex, and cerebellum (Monsivais et al., 2024). Hypothesized mechanisms of toxicity include impaired calcium homeostasis (Ijomone et al., 2019), mitochondrial dysfunction (Martinez-Finley et al., 2013), neuroinflammation,(Harischandra et al., 2019) altered proteostasis, (Harischandra et al., 2019) impaired microRNA function, (Wallace et al., 2020) and altered metabolism of neurotransmitters including ACh (Soares et al., 2020). Mechanistically, animal and molecular models demonstrate the diffuse effects of Mn on multiple areas of the neuronal cholinergic synapse that could contribute to cholinergic dysfunction. Mn inhibits presynaptic striatal and extrastriatal choline uptake in the caudate, putamen, hippocampus, frontal and parietal cortices, (Eriksson et al., 1984) and once within the nerve terminal, Mn appears to have direct effects on the quantal release of acetylcholine into the synaptic cleft (Kita et al., 1981; Glitsch and Pott, 1978). Further, Mn has regional and temporal effects on the degradation of acetylcholine (ACh) by regulating acetylcholinesterase (AChE) activity (Santos et al., 2012; Zhang et al., 2001). Interestingly, Mn decreases striatal and midbrain presynaptic choline acetyltransferase (ChAT) activity in the striatal cholinergic terminals of chronically exposed rats (Lai et al., 1981). In our study, we identify a similar dose-dependent association between VAT binding of VAChT (which is tightly regulated with ChAT) in the caudate and cortical regions of Mn-exposed workers in association with higher levels of cumulative Mn exposure.

Additionally, we identified that lower cholinergic activity underlies a component of the relationship between higher Mn exposure and lower cognitive control including lower performance on the VF task. These findings are consistent with our previous studies in which cognitive control performance was related to environmental and occupational Mn exposure (Al-Lozi et al., 2017; Racette et al., 2022) and the larger literature documenting more generalized cognitive deficits including, but not limited to visual spatial skills, working memory, cognitive flexibility, attention, and concentration in both human (Bowler et al., 2003; Bowler et al., 2006; Park et al., 2009; Park, 2013; Vlasak et al., 2023) and controlled animal models (Schneider et al., 2015; Schneider et al., 2013; Newland and Weiss, 1992; Burton and Guilarte, 2009; Smith and Strupp, 2023; Kern et al., 2010). Cognitive control encompasses the goal-oriented ability to manipulate information, to distinguish and identify relevant data, to be flexible, and to self-regulate to achieve high order objectives including adaptation, prioritization, and organization (Botvinick and Braver, 2015; Rabinovici et al., 2015; Al-Lozi et al., 2017). Identifying deficits in these areas has direct relevance to workers and exposed individuals as impairment in these domains could have detrimental effects on work performance and daily function. Further, cholinergic neurons are physiologically essential in locomotion and cognition (Finkelstein et al., 2007) and gradual age-associated loss of cholinergic function has been implicated in age-related functional decline and cognitive impairment (Schliebs and Arendt, 2006). Severe loss of cholinergic neurons ultimately results in clinical dementia (Schliebs and Arendt, 2006). Here we demonstrate exposure to Mn relates to lower cholinergic activity and poorer cognitive control/individual VF performance, above and beyond the expected effects of age. Longitudinal investigations will be necessary to see if this effect progresses to include deficits on individual memory tasks and increases the risk for age-related functional decline and/or neurodegeneration including dementia.

In this study, cumulative Mn exposure was strongly related to lower cholinergic activity in the caudate, precuneus, pars triangularis, pars opercularis, middle temporal lobe, and entorhinal cortex. The caudate, in particular, has been implicated in the pathophysiology of Mn as an area demonstrating higher T1 MRI signal (Mn deposition) and dopaminergic dysfunction (Criswell et al., 2018; Criswell et al., 2012). Similarly, as part of the mesocortical dopaminergic circuit, the caudate is also heavily implicated in cognitive control dysfunction associated with PD (Hirano, 2021; Bohnen et al., 2015). However, while striatal dopaminergic denervation is common even in PD patients without any cognitive impairment, the presence of cortical cholinergic denervation is more specific to those with cognitive deficits (Bohnen et al., 2015, 2006) demonstrating the complex functional interactions between anatomically distinct regions within the brain’s dopaminergic and cholinergic networks. The dual presence of striatal dopamine and cortical cholinergic denervation in PD patients with cognitive impairment highlights the complex, multi-system, multi-transmitter deficiencies likely present in PD and related parkinsonian conditions. The caudate nucleus contains a large number of cholinergic interneurons which are involved in attention, goal-directed actions, habit formation, and behavioral flexibility through modulation of all striatal cell types including D1R and D2R medium spiny neuron (Pisani et al., 2007; Bohnen and Albin, 2011). However, little is known about interneuron behavior in PD and this may represent an important area of future exploration. Our findings demonstrating lower cholinergic activity in association with cognitive dysfunction in Mn neurotoxicity, also emphasize the need to study non-dopaminergic neurotransmitters when investigating basal ganglia mediated cognitive control dysfunction (Mckeown and Peavy, 2015). Future, multi-modal studies including multiple neurotransmitters may represent complementary biomarkers of both motor and cognitive deficits associated with Mn exposure and provide a better mechanistic understanding of pathophysiology of Mn neurotoxicity and parkinsonian syndromes.

## 5. Limitations

This study has several potential limitations. Welding fume contains multiple gases and elements, including iron, lead, chromium and nickel; (NIOSH, 1988; Abdullahi and Sani, 2020; Li et al., 2004) therefore, we cannot fully exclude a contribution from other potential welding fume neurotoxicants. Our study relies on Mn exposure estimates based on work histories; however, we observed a similar pattern of results with the T1 MRI PI. Our sample size is limited in this study, but we observed effects, nonetheless. The models we specified make assumptions about linearity in the inter-relationships between Mn exposure, age, cholinergic VAT BP_ND_, and cognitive control performance. These assumptions were generally supported by the data, but this approach will not capture non-linear relationships. We also did not consider interactions between our variables or potential confounders other than age. To make our mediation findings more statistically robust, we leveraged bootstrapping techniques. This approach did not depend on sample-wise assumptions of normality for claims of statistical significance. Another consideration is that for mediation analysis, we leveraged regions with Mn exposure and VAT BP_ND_ associations, without incorporating cognition into our region selection. We feel this approach is justified as the regions with the highest propensity to be affected by Mn exposure would likely also have strong associations with cognitive control dysfunction and would best demonstrate a directed path from Mn exposure to altered cognition control ability. Finally, this is a cross-sectional study examining the cholinergic system via [(18)F]VAT PET and, thus, does not address whether Mn induces progressive cognitive decline which would be suggestive of a neurodegenerative phenotype. Future longitudinal studies including serial cognitive evaluations in conjunction with multi-radioligand PET studies, may be helpful in further elucidating the underlying mechanisms of Mn neurotoxicity.

## 6. Conclusion

In summary, we demonstrated that higher Mn exposure relates to lower cholinergic activity in the caudate, the precuneus, pars triangularis, pars opercularis, middle temporal lobe, and entorhinal cortex, and lower cholinergic activity in these regions mediates a portion of the relationship between Mn exposure and cognitive control function. Caudate and cortical cholinergic activity may be of particular interest as a biomarker of Mn neurotoxicity and key regions in understanding the underlying mechanism of cognitive control dysfunction in parkinsonian syndromes including Mn neurotoxicity, PD, and PDD.

## Figures and Tables

**Table 1:** Participant characteristics.

Characteristic	N = 21 n (%)
**Sex**	
Male	19 (90.4)
Female	2 (9.6)
**Race and ethnicity**	
Non-Hispanic White	21 (100)
**Education**	
Graduated High School/GED	17 (80.95)
Two Years of Higher Education	4 (19.05)
**Age, years**	
Mean (SD)	53.95 (13.95)
Min	31
Median	58
Max	73
**Duration Mn exposure, years**	
Mean (SD)	13.43 (13.72)
Min	1.25
Median	4.95
Max	41.09
**Cumulative Mn exposure, mg Mn/m** ^**3**^ **-years**	
Mean (SD)	1.88 (1.92)
Min	0.17
Median	0.69
Max	5.75
**Pallidal Index**	
Mean (SD)	112.07(4.56)
Min	103.28
Median	112.47
Max	121.91

SD = standard deviation; Mn = manganese.

**Table 2:** Cognitive control performance in Mn-exposed workers.

	N	Mean	Min	Median	Max
**VF Scaled Score** ^a^	18	8.78	1.00	9.50	14.00
**2B Discriminability** ^b^	17	0.52	−0.22	0.57	0.81
**LNS Scaled Score** ^a^	18	10.11	6.00	9.00	16.00
**GnG Discriminability** ^c^	18	0.91	0.66	0.92	1.00
**Simon Reaction Time Interference Effect** ^d^	18	43.90	2.00	50.13	85.75
**Cognitive Control Summary Score** ^e^	17	0.14	−4.20	−1.00	5.60

Mn = manganese; VF = Verbal Fluency; 2B = Two-Back Letter Task; LNS = Letter Number Sequence; GnG = Go-No-Go.

a Age-adjusted total scaled score scaled to normative data.

b Discriminability is defined as mean accuracy rate minus the false positive response rate.

c Difference between accuracy and false alarm rate

d The difference between the reaction time for congruent and incongruent reaction time was used.

e Mean of the z-scores of each of the five tests.

**Table 3:** VAT BP_ND_ mediation between Mn exposure and cognitive control performance.

		Median β	90 % CI	
**VF Scaled Score**	mg Mn/m^3^-years	−0.9443*	−2.1574	−0.0651
	Age (years)	−0.0037	−0.0430	0.0224
	Total	−0.9553*	−2.1554	−0.0655
**2B Discriminability**	mg Mn/m^3^-years	−0.0161	−0.0762	0.1121
	Age (years)	−0.0002	−0.0025	0.0005
	Total	−0.0163	−0.0770	0.1114
**LNS Scaled Score**	mg Mn/m^3^-years	−0.2186	−1.1219	0.6210
	Age (years)	−0.0002	−0.0145	0.0193
	Total	−0.2203	−1.1024	0.6253
**GnG Discriminability**	mg Mn/m^3^-years	−0.0089	−0.0704	0.0156
	Age (years)	0.0000	−0.0004	0.0012
	Total	−0.0089	−0.0696	0.0159
**Simon Reaction Time Interference Effect**	mg Mn/m^3^-years	−2.6138	−22.7866	7.4858
	Age (years)	−0.0117	−0.2589	0.1619
	Total	−2.6648	−22.7219	7.4576
**Cognitive Control Summary Score** ^a^	mg Mn/m^3^-year	−0.661*	−2.130	−0.032
	Age (years)	−0.002	−0.031	0.026
	Total	−0.671*	−2.113	−0.032

Median β is taken from 5000 bootstrapped β estimates; Coefficients are not comparable across individual tests.

* indicates significant mediation (CI excludes 0).

a Mean of the z-scored VF, 2B, LNS, GnG, and Simon tests.

BP_ND_ = non-displaceable binding potentials; Mn = manganese; CI = confidence interval; VF = Verbal Fluency; 2B = Two-Back Letter Task; LNS = Letter Number Sequence; GnG = Go-No-Go.

## Data Availability

Data will be made available on request.
